# A hybrid approach toward biomedical relation extraction training corpora: combining distant supervision with crowdsourcing

**DOI:** 10.1093/database/baaa104

**Published:** 2020-12-01

**Authors:** Diana Sousa, Andre Lamurias, Francisco M Couto

**Affiliations:** LASIGE, Departamento de Informática, Faculdade de Ciências, Universidade de Lisboa, Lisboa 1749-016, Portugal; LASIGE, Departamento de Informática, Faculdade de Ciências, Universidade de Lisboa, Lisboa 1749-016, Portugal; LASIGE, Departamento de Informática, Faculdade de Ciências, Universidade de Lisboa, Lisboa 1749-016, Portugal

## Abstract

Biomedical relation extraction (RE) datasets are vital in the construction of knowledge bases and to potentiate the discovery of new interactions. There are several ways to create biomedical RE datasets, some more reliable than others, such as resorting to domain expert annotations. However, the emerging use of crowdsourcing platforms, such as Amazon Mechanical Turk (MTurk), can potentially reduce the cost of RE dataset construction, even if the same level of quality cannot be guaranteed. There is a lack of power of the researcher to control who, how and in what context workers engage in crowdsourcing platforms. Hence, allying distant supervision with crowdsourcing can be a more reliable alternative. The crowdsourcing workers would be asked only to rectify or discard already existing annotations, which would make the process less dependent on their ability to interpret complex biomedical sentences. In this work, we use a previously created distantly supervised human phenotype–gene relations (PGR) dataset to perform crowdsourcing validation. We divided the original dataset into two annotation tasks: Task 1, 70% of the dataset annotated by one worker, and Task 2, 30% of the dataset annotated by seven workers. Also, for Task 2, we added an extra rater on-site and a domain expert to further assess the crowdsourcing validation quality. Here, we describe a detailed pipeline for RE crowdsourcing validation, creating a new release of the PGR dataset with partial domain expert revision, and assess the quality of the MTurk platform. We applied the new dataset to two state-of-the-art deep learning systems (BiOnt and BioBERT) and compared its performance with the original PGR dataset, as well as combinations between the two, achieving a 0.3494 increase in average *F*-measure. The code supporting our work and the new release of the PGR dataset is available at https://github.com/lasigeBioTM/PGR-crowd.

## Introduction

Knowledge bases play a fundamental role in the way we store, organize and retrieve information. More specifically, biological knowledge bases are commonplace for researchers and clinicians to access all types of biomedical data retrieved from the biomedical literature (1). Previous works annotated the biomedical literature by resorting to domain expert annotators (2), crowdsourcing platforms (3) or distantly supervised techniques (4). These researchers’ main aim was to tackle the lack of annotated datasets for biomedical information extraction systems. However, when applying distantly supervised techniques, the annotations are not as reliable as when done by domain experts, and it still needs to be adequately reviewed before the extracted information can be added to any biomedical repository. Hence, the added advantage of automating information extraction using distant supervision is slightly impaired by the need to review it, which is costly and time- and resource-consuming. Moreover, when targeting relation extraction (RE) between entities of different domains or document summarization tasks (5), the revision process becomes cumbersome compared with other information extraction tasks, given its higher complexity that usually requires knowledge of multiple domains.

The alternative way to create reliable gold standard datasets that do not resort to domain expert curation could be allying distant supervision with crowdsourcing (6–8). Before integrating the data extracted from distant supervision pipelines into biological knowledge bases or using it as training data for biomedical information extraction systems, the data would go through a confirmation or review phase in the form of crowdsourcing. Crowdsourcing platforms are becoming increasingly popular to address the lack of training corpora for natural language processing (NLP) tasks (9). The most popular platform for this purpose is Amazon Mechanical Turk (MTurk) (10–12). Some platforms created a trust layer over MTurk to facilitate task specification and monitoring (13), such as Figure Eight Inc. company (previously known as CrowdFlower) (14, 15), which is widely used by researchers for biomedical NLP-related tasks.

One of the problems of using crowdsourcing platforms is the lack of domain expertise. While most platforms allow us to specify some criteria (e.g. degree of education), in exchange for an increased price per task, it is not feasible to specify expertise in particular biomedical domains. Not only that, but there is no guarantee that the quality promised is the quality provided because some malicious workers often take advantage of the difficulty in implementing a verification procedure and submit answers of low quality (9). Task redundancy can be a solution, but it also increases the costs of using crowdsourcing approaches, partially defeating the purpose of these platforms. The question should be whether the workers’ quality is good enough for the purpose of the task and if the decrease in costs compensates the difference in quality compared with domain experts. In the case of the MTurk platform, some studies have supported its suitability for a variety of tasks (16). However, it fails in transparency about its workers’ context (e.g. background), if MTurk constitutes their primary form of income or not, what is their motivation for completing the tasks and if this introduces bias to the tasks at hand. These and other ethical questions have been discussed in depth by some researchers (17, 18).

Previous works have combined distant supervision with crowdsourcing, specifically for non-biomedical relations. Gormley et al. (6) present an approach that allies distant supervision with MTurk crowdsourcing for relations between nominals (e.g. places and persons). Liu et al. (7) used a gated instruction (GI) protocol to perform crowdsourcing on person–location relations, building their own interface. The GI protocol trains the workers to annotate a sentence while providing motivational feedback, removing workers who do not meet with a pre-defined reputation threshold at the end of the first stage of training. Collovini et al. (8) used a pre-existing Portuguese nominal relations dataset to perform crowdsourcing with Figure Eight Inc. company with the primary goal of expanding Portuguese annotated data. However, none of these approaches assessed the validity of their revised datasets beyond worker statistics. Also, there is a lack of approaches targeting the biomedical domain, which is inherently more complex.

In this work, we leveraged an existing dataset of biomedical relations, created through distant supervision, and submitted it to the MTurk platform to perform crowdsourcing validation. With the exhaustive review of the original and new datasets’ performance, we assessed the viability of combining distant supervision and crowdsourcing for the field of biomedical RE.

Our work used an open-source dataset, the phenotype–gene relations (PGR) dataset (4), based on distant supervision, that features both human phenotype and gene annotations and their relations. Some researchers already used the PGR dataset as training data (19–21) while others opt out of using it for being a silver standard (22). Since it is a silver standard dataset, domain experts have not reviewed it, leading to wrongly labeled relations and other errors. These errors can be from named-entity recognition (NER) (e.g. acronyms of diseases annotated as genes), which was also done automatically, or sentence format errors. To rectify these errors, we used the MTurk platform to validate, alter or discard the PGR dataset’s relations. We achieved this by dividing the original dataset into two partitions, one of 70% (Task 1), where each relation was rated by one Amazon worker, and another of 30% (Task 2), where each relation was rated by seven distinct workers. We validated our approach through inter-rater agreement using the Fleiss’ kappa (23) and the Krippendorff’s alpha (24) metrics for Task 2. Further, we also provided the 30% partition of the PGR dataset used for Task 2 to an external rater (on-site, without previous curating experience but holding a biochemistry degree) and a domain expert (with previous curating experience, holding a PhD in bioinformatics). These different levels of expertise enlightened the difficulties of curating the dataset and the limitations associated with each level. To evaluate and compare the quality of the crowdsourced Amazon dataset, we applied it to two state-of-the-art deep learning systems and compared its performance with the original PGR dataset, as well as combinations between the two. The deep learning systems used were BiOnt (25) and BioBERT (26), which feature RE between different biomedical entities with high performance, and, in the case of BiOnt, it was already used in conjunction with the PGR dataset.

The MTurk workers’ performance compared with our on-site curator and the domain expert was generally good for accessing NER or sentence format errors (∼16% of relations). However, the MTurk workers struggle to identify false relations (separate entities with no association in a sentence). The struggle to identify these relations can be due to the complexity of the sentences or quality issues related to the MTurk platform validation of workers, which we will discuss in more detail in the following sections. Further, the inter-rater agreement for Task 2 showed a slight to a fair agreement (∼0.20–0.21), which is below what we expected, and we believe it could be related to the problems of sentence complexity and quality reported. Regarding the performance of the crowdsourced Amazon dataset in applying the BiOnt and BioBERT systems, we had an increase in average *F*-measure of 0.3494, taking into account all the experiences concerning the original PGR dataset.

The main takeaways of this work were the need for further validation of the use of crowdsourcing platforms, such as the MTurk platform, and the potential of using distant supervision allied with crowdsourcing to produce gold standard datasets with which we can train viable models and detect relevant biomedical relations. This work resulted in the following contributions:

- Pipeline for RE crowdsourcing, in which we describe in detail all the base concepts and steps taken to produce the new crowdsourced dataset.
- New release of the PGR dataset, which will be made freely available to the community.
- Assessment of the quality of results obtained with the MTurk platform (through statistical analysis and direct comparison with on-site rater and domain expert).

## Materials and methods

This section presents an overview of the PGR dataset (4), a brief presentation of the Amazon MTurk platform and the integration of the dataset into the MTurk platform (including the design, configuration and evaluation stages). We now describe how we proceeded with each of these stages:

- Design
  (a) Set up the tasks (human intelligence task—HIT) to be simple to understand and easy to accomplish by the employees (i.e. workers or turkers).
  (b) Define the guidelines (instructions) with examples for the workers to better understand the presented HITs.
- Configuration
  (a) Configure the MTurk platform specifying different criteria (for workers) and wage (i.e. reward).
  (b) Submit the HITs within the platform.
- Evaluation
  (a) Calculate the inter-rater agreement.
  (b) Compare the PGR dataset before and after MTurk crowdsourcing assessment by employing two different deep learning models (BiOnt (25) and BioBERT (26))

**Figure 1.**
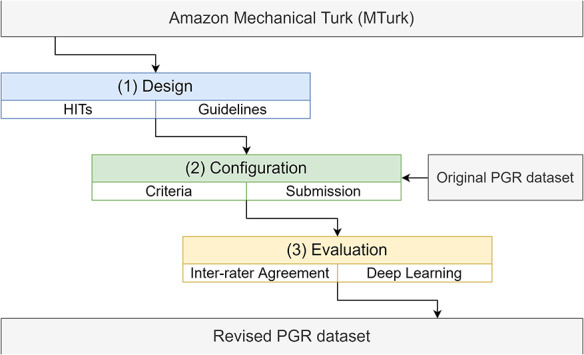
The pipeline to incorporate the PGR dataset into the Amazon MTurk platform, including the design, configuration and evaluation stages.

An overview of the pipeline of the work described in this paper is shown in Figure 1.

### PGR dataset

The PGR dataset is a silver standard corpus of PubMed abstracts featuring human phenotype and gene annotations and their relations (4). In this dataset, all the annotations were generated in a fully automated fashion (silver standard), taking a distant supervision approach, opposite to a manually annotated dataset where domain experts generate the annotations (gold standard).

The first release of the PGR dataset focused mostly on the initial release of the dataset (10/12/2018). A small subset of relations (6%) was manually reviewed to evaluate the PGR dataset quality and use as a test corpus for machine learning model evaluation. The second release (11 March 2019) captured a more clear-cut search of the type of abstracts to retrieve, such as abstracts regarding diseases, their associated phenotypes and genes, increasing from ∼2.5 relations per abstract to ∼3.0 relations per abstract, and the overall number of relations by 2-fold. In this work, we will use the second release of the PGR dataset to generate an improved third release.

**Figure 2.**
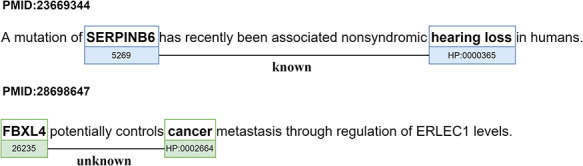
Examples of the two types of relations (‘Known’ and ‘Unknown’) in the PGR dataset (partial figure from (28)). The sentence of abstract PMID:23 669 344 was simplified to capture more clearly the ‘Known’ relation.

The relations identified in the PGR dataset are either ‘Known’ if present in the knowledge base of relations provided by the human phenotype ontology (HPO) group (27) or ‘Unknown’ otherwise. Figure 2 presents examples of the two types of relations (‘Known’ and ‘Unknown’). Figure 2 also displays the most prominent problem in silver standard datasets. The ‘Unknown’ relation is marked as false due to the relation between the *FBXL4* gene and the human phenotype ‘cancer’ not being represented in the gold standard knowledge base, even though it is true.

Table 1 presents the numbers for the second release of the PGR dataset.

**Table 1.** The number of abstracts, phenotype and gene annotations, and known, unknown and total relations for the second release (11 March 2019) of the PGR dataset (partial table from (4))

	Annotations		Relations		
Abstracts	Phenotype	Gene	Known	Unknown	Total
2657	9553	23 786	2480	5483	7963

### Amazon MTurk

The Amazon MTurk is a crowdsourcing web service (marketplace) that facilitates the use of human intelligence to individuals and businesses that are in demand to complete specific tasks (29). In this web service, the employees (i.e. workers or turkers) execute tasks (i.e. HITs) submitted by employers (i.e. requesters) to earn a pre-defined wage (i.e. reward). The type of HITs that MTurk allows requesters to submit ranges from sentiment analysis and document classification in the language domain to image classification in the vision domain. Requesters post-HITs to workers who meet their specified criteria (e.g. degree of education), and pre-defined both a reward and maximum time allotted to complete each task. Both requesters and workers remain anonymous throughout the process (workers can be identified through Amazon’s internal identifier).

The three main benefits of the MTurk platform are as follows: (i) optimized efficiency by allowing requesters to outsource tasks that need to be handled manually, but do not require the requester or their employees’ expertise; (ii) increased flexibility for requesters to quickly scale their businesses without needing to scale their in-house workforce and (iii) cost reduction by eliminating the need for requesters to employ a temporary workforce and all the management costs associated with it (10).

Some previous works using MTurk in the biomedical field include NER and curation of biomedical entities labels. Yetisgen-Yildiz et al. (11) used MTurk to extract named entities such as medical conditions, medication and laboratory tests, from clinical trial descriptions. Good et al. (30) used it for disease mention annotation in PubMed abstracts. Similarly to our approach, Khare et al. (12) used MTurk to curate indications from drug labels, i.e. to judge whether a drug is used in managing a highlighted disease. In particular, with medical corpora, MTurk was also used to validate medical information shared on Twitter (31) and classify medical notes relevant for a particular subject (e.g. diabetes) (32). Further, researchers used MTurk with electronic health records (EHR), for instance, to identify mentioning of abnormal ear anatomy in radiology reports (33), to validate the simplification of EHR for patients (34) and as a preprocessing step to create data for autism detection systems (35), among other applications.

### Integration into Amazon MTurk platform

The MTurk platform provides a wide range of customizable templates to start a new project. The template closest to our previously described curation task was the document classification template, within the language field, that we leveraged to set up our PGR HITs. To facilitate the evaluation of the workers’ performance, we divided the original dataset into partitions of 70% (Task 1), where each relation was rated by one Amazon worker and 30% (Task 2), where each relation was rated seven times, by seven distinct workers. We also had to define guidelines (instructions) with examples for the workers to understand the task at hand thoroughly. Further, each project required defining criteria to select the workers that better suited the project’s goals and determining the reward per HIT for each worker before submission. Finally, after receiving the results (which took about two weeks), we had to evaluate our workers’ performance. The evaluation was done by calculating the inter-rater agreement and comparing the PGR dataset’s performance before and after curation with existing deep learning tools.

We describe the detailed steps we took and the reasoning for each decision in the following sections.

### Design

#### HITs

**Figure 3.**
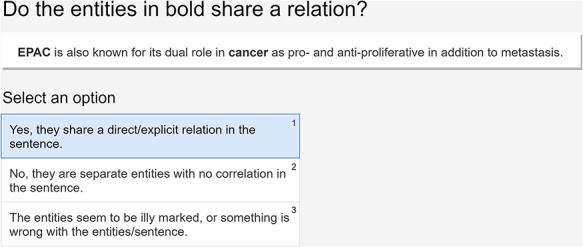
An example of a HIT presented to the workers and the available options.

As stated previously, we adapted the document classification template to set up our HITs. Thus, the workers were presented with a sentence with two entities in bold (the human phenotype and the gene entities) and a set of three possible classifications (true relation, false relation or wrongly labeled relations due to errors in the NER stage or wrong sentence format). Figure 3 represents an example of a HIT as presented to the workers (Task 2).

#### Guidelines

In this work, we considered that rather than defining strict guidelines, it would be more intuitive for the workers to be presented with examples of instances and their gold labels (Supplementary Material Figure 1). Nonetheless, the primary goal of the task presented to the workers was ‘to choose among three possible options to classify the relation between a phenotype and a gene in each sentence’. The guidelines presented to the workers are illustrated in Supplementary Material Figure 1. We opted out of more exhaustive guidelines to keep the task time manageable and more straightforward to understand. Adding an option to each HIT that expressed decision difficulties of the workers was considered by our team. However, eventually, we feared that it would become the default option, given the complexity of most sentences.

As we do not have access to tools that validate each worker through MTurk (e.g. to determine if they are of malicious intent), we validated our guidelines by launching a small subset of 10 sentences. These sentences served exclusively to validate the approach, although we recognize that more extensive guideline testing could be performed with more financial resources and more validation functionalities by MTurk.

### Configuration

#### Criteria

As we stated before, requesters can pre-define specific criteria that the workers have to meet to work on a task. However, specifying that criteria have an added cost per HIT that would make the total value for the task too expensive, invalidating the use of the crowd (domain expertise would be about the same value). Therefore, the criteria chosen and the cost of the crowdsourcing project described in this work are detailed in Table 2. The requirement that workers be ‘Masters’ (high-performing workers according to MTurk) adds $0.001 to the MTurk fee, but since the platform rounds it up to the cent, the total value is unaltered.

**Table 2.** Summary of the crowdsourcing task criteria and associated costs

Setting	Task 1	Task 2
Reward per assignment (USD)	0.02	0.02
MTurk fee (USD)	0.01	0.01
Number of assignments per task	1	7
Minimum time per assignment	3s	3s
Require that workers be masters to do your tasks (high-performing workers according to MTurk)	Yes	Yes
Number of tasks	5574	2389
Total cost (USD)	167.22	501.69

We opted for this distribution of assignments, one for Task 1 and seven for Task 2, due to budget constraints. Ideally, we would like to have seven assignments for both tasks to fully evaluate the impact and differences of having multiple workers rating each HIT. However, considering these constraints, we considered that having a focused task, such as Task 2, with seven assignments for 30% of the dataset (i.e. an odd number to facilitate consensus) would be more relevant than having fewer judgments per relation. The number seven was chosen as a better guarantee of quality to reduce the impact of lower-quality annotations. Using a lower odd number such as three or five, an annotation at random or malicious would be more detrimental for the final assignment. A higher number would necessarily implicate a higher cost and would not necessarily add significant quality benefits to the task, as stated by Kappel et al. (36) and Cooke et al. (37).

#### Submission

We designed a web page template for the tasks and defined the project properties, as required by the MTurk platform. We provided the input instances as a CSV file, where each line corresponded to a HIT. Alternatively, platforms such as Figure Eight Inc. company (12) simplify task specifications and monitor MTurk tasks. However, we worked directly with the MTurk platform.

### Evaluation

#### Inter-rater agreement

The original dataset was divided into 70%, where each relation was rated by one Amazon worker and 30%, where each relation was rated seven times by seven distinct workers. The goal of rating a subset of relations with overlap (Task 2) was to assess if the raters agreed with each other about the exact rating to be attributed (among the three previously described), by measuring the inter-rater agreement. To determine the last metric, we used both the Fleiss’ kappa (23) and the Krippendorff’s alpha (20) metrics appropriate for nominal ratings. The Fleiss’s kappa metric is a statistical measure that estimates the reliability of agreement between a fixed number of raters, assuming that our raters were chosen at random from a larger population. Similarly, Krippendorff’s alpha is a statistical measure of the agreement, useful when we have multiple raters and multiple possible ratings. We opted by using the two metrics to validate our work. A low deviation between the two metrics will assure an unbiased estimate (38). Furthermore, we added an additional rater from our research center with no previous curating experience, but with a strong background in biochemistry, to rate the overlapping subset of relations. The cost of this addition was $1247 for the rating of the 2389 relations in Task 2. This additional rater was fundamental to understanding the challenges that our workers faced and improving our curation pipeline and guidelines in the future. The extra rater took one month to complete Task 2.

**Figure 4.**
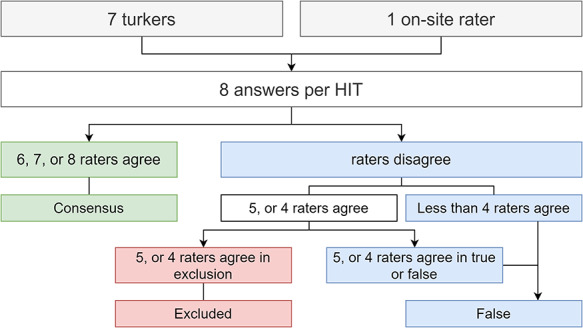
Flowchart illustrating how to reach majority consensus, according to the answers provided by the workers plus our extra rater on-site.

To reach a majority consensus between the workers (for Task 2), we used a voting scheme, similar to the approach of Shu Li et al. (14). According to the voting scheme, Figure 4 illustrates how we chose to classify a relation true, false or be excluded. We considered that if at least half of the answers voted to exclude the relation from the dataset, the relation should be excluded. Our default label was false because we considered that false relations are more challenging to assess; hence, if a worker is in doubt between true and false, the most likely label would be false. For example, if on one HIT five out eight raters agreed to exclude, we accepted that rating. However, if five agreed true or false, we classified it as false, since considering it a valid sentence (not to exclude), with no agreement, our default label is false. The false label is also a safer option in our target domain. Having a true relation that is, in fact, false (false positive) is more detrimental for the learning process than a false negative. We could have considered using the amount of time spent on each question by the workers to build a voting schema, but each HIT only took a few seconds to complete. Thus, because each HIT only takes a few seconds, other worker factors could be at play beyond difficulties in rating a specific sentence. These factors can be a slower Internet connection, language barrier, or even just taking a break mid-work.

To further assess the quality and challenges of curating the PGR dataset and validate the previous approach, a domain expert with a bioinformatics background and experience in using and curating corpora also curated Task 2. This domain expert was an in-house researcher. Therefore, there was no direct cost associated with his curation task, although we could extrapolate that would be at least the same as the extra rater. The same guidelines provided to the Amazon workers were provided to the external rater (on-site), which did not access external information. The domain expert had access to external information as needed. The external rater (on-site) could contact the expert for further elucidations on some of the HITs. The domain expert took 2 weeks to complete Task 2.

Not only the extra rater and domain expert ratings took longer to obtain, but they also were more expensive. To consider that their ratings are more worthwhile than MTurk workers’ ratings, these have to surpass them in performance when applying to RE deep learning systems. Even then, we have to ponder cost and time and how much that affects the evaluation metrics for it to be worth choosing one route over the other. Introducing more information to MTurk workers, as was provided to the domain expert, is a route that we did not explore due to the necessary increase in the cost per HIT. More information would take more time to be processed. Therefore, the payment for each HIT had to be increased, invalidating the cost-benefit of using MTurk in detriment of domain expertise.

#### Deep learning systems

To further access the quality of the crowdsourced curated dataset, we applied it to two distinct deep learning systems that target the biomedical domain: BiOnt (25) and BioBERT (22).

The BiOnt system is a deep learning system based on the BO-LSTM system (39) used to extract and classify relations via long short-term memory networks and biomedical ontologies. This system detects and classifies 10 types of biomedical relations, such as human PGRs. It takes advantage of domain-specific ontologies, like the HPO (27) and the gene ontology (GO) (40). The BiOnt system represents each entity as the sequence of its ancestors in their respective ontology. To create our models, we used the default parameters indicated in the original research. The relevant configurations for model training were the mini-batch gradient descent optimization algorithm (RMSprop), learning rate (0.001), loss function (categorical cross-entropy) and dropout rate (0.5 for every layer except the penultimate and output layers).

The BioBERT system is a pre-trained biomedical language representation model for biomedical text mining based on the BERT (41) architecture. This system can perform diverse biomedical text mining tasks, namely NER, RE and question answering, when trained on large-scale biomedical corpora. The architecture’s novelty is that their authors designed these systems (BioBERT and BERT) to pre-train deep bidirectional representations by jointly conditioning on both left and right context in all layers. This feature allows easy adaption to several tasks without loss in performance. The default relevant parameters for BioBERT are the same as for BERT. The configurations for model training were the mini-batch gradient descent optimization algorithm (Adam), learning rate (1e-4) and dropout probability (0.1 on all layers). The training loss is the sum of the mean masked language model likelihood and the mean next sentence prediction likelihood.

For comparison, we tested both the original PGR dataset (second release, Table 1) and the crowdsourced Amazon dataset and combinations between the two (detailed in Table 6). We primarily used the ‘Amazon Task 1’ data as training data and the ‘PGR original test set’, the ‘Amazon/extra-rater consensus Task 2’ data and the ‘expert Task 2’ data as test data. We also made combinations between the two tasks using the ‘Amazon/extra-rater consensus Task 2’ data and the ‘expert Task 2’ data as training data, and the ‘PGR original test set’ as test data. It is necessary to point out that the ‘PGR original test set’ refers to the first release of the dataset (since the second release did not have a test set), so there is no overlap between the datasets used for training and testing.

## Results and discussion

### Ratings statistics

**Figure 5.**
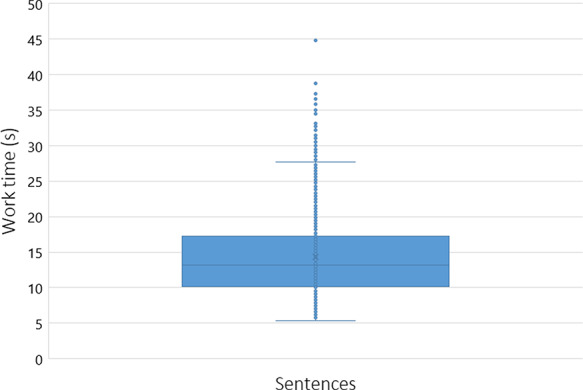
Box plot expressing the average worker work time distribution (in seconds) per sentence (with a cutoff of 50 seconds).

**Figure 6.**
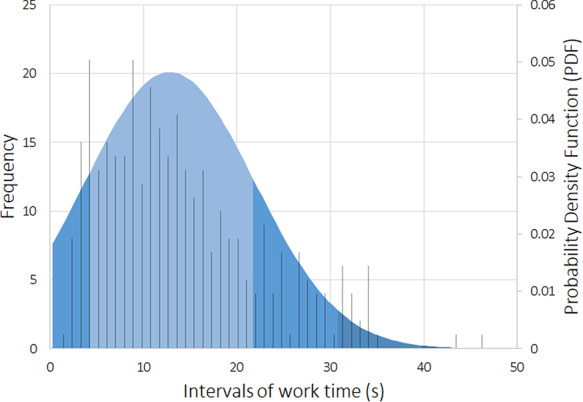
Standard deviation expressing the average worker work time distribution (in seconds), and the histogram of the occurrence events (with a cutoff of 50 seconds).

To assess the workers’ performance, we conducted some statistical analyses, including the time spent on average rating each sentence. Figures 5 and 6 reflect the workers’ average time with each sentence, with a cutoff of 50 seconds (using box plot and standard deviation analysis). We decided to set the cutoff for work time to 50 seconds because we considered that as enough time for a worker to make an assessment, and anything longer than that was probably the worker having a mid-task break (the longest time for a HIT completion was 40 322 seconds, ∼11 hours). Thus, we had multiple HITs that lasted well above 50 seconds. However, to paint a clearer picture of most responses, we limited the statistical analysis to the workers that took <51 seconds to complete a HIT. Therefore, we did not impose a time limit for completing a HIT not to constrain workers to have to rush their decisions.

Our domain expert did a similar time self-evaluation, which resulted in an average of ∼20 seconds per sentence (for Task 2). The domain expert consulted some abstracts to clarify whether an abbreviation referred to a gene or other type of entity for a specific sentence. Through Figures 5 and 6, it is possible to assess that workers took an average of 13 seconds per HIT (sentence). By comparing this time to the average time done by our domain expert (20 seconds), it is possible to question the level of attention with which our workers performed their ratings, questioning the trust that we can deposit on MTurk crowdsourcing. However, considering that our domain expert took some time checking some abstracts to which workers did not have access, it can justify the differences in average time. We considered providing the same access to the workers, but that would invariably make them spend more time on each HIT, raising the cost of each HIT and the overall task. In those conditions, even if we considered paying $0.10 ($0.09 + $0.01 MTurk fee) as a fair reward per assignment instead of the $0.03, that would raise our total budget >3-fold, invalidating the cost-benefit of using MTurk in detriment of domain expertise.

To further characterize the workers that performed our tasks, we checked their WorkerId tab in the results file provided by MTurk. There, we realized that six sentences were rated but did not have an associated WorkerId. Both tasks (7983 relations, 22 255 HITs) were performed by only 64 different workers, making 348 HITs per worker. Therefore, if we had a malicious worker that classified their respective HITs at random or close to it, it would damage the whole dataset. The MTurk platform should guarantee a more diverse group of workers working on the same task since that is what employees expect, even to avoid some bias ratings or a more strict selection process.

### Inter-rater agreement

Table 3 presents the inter-rater agreement score, using both Fleiss’ kappa (23) and Krippendorff’s alpha (24) metrics, for the dataset corresponding to Task 2, considering only the Amazon workers, the Amazon workers plus the extra rater (on-site) and the extra rater (on-site) plus the domain expert.

**Table 3.** The inter-rater agreement score, using both Fleiss’ kappa and Krippendorff’s alpha metrics, considering only the Amazon workers, the Amazon workers plus the extra rater (on-site) and the extra rater (on-site) plus the domain expert (Task 2)

	Inter-rater agreement		
Inter-rater agreement metric	Amazon workers	Amazon workers + extra rater (on-site)	Extra rater (on-site) + expert
Fleiss’ kappa	0.2028	0.2050	0.6549
Krippendorff’s alpha	0.2029	0.2051	0.6550

The number of different workers classifying the senten-ces on Task 2 (33) reflects that workers rated, on average, 77 different sentences. Although for each set of seven identical sentences, MTurk guarantees unique workers, there is no guarantee that a worker works on all sentences or that a worker only rates one sentence. Ideally, we would like to have seven workers working on all the sentences, or non-repeating workers for all ratings. Given this small number of workers working on Task 2 and the high number of sentences to rate (2389), it is challenging to find an inter-rater agreement metric that can return an accurate value of agreement between the workers. The Fleiss’ kappa metric assumes that the raters are deliberately chosen and fixed, while the Krippendorff’s alpha metric is indicated for when we have multiple raters and multiple possible ratings. Since none of the two cases is precisely right, we do not have a metric that fully expresses our experiment’s results with Task 2. We can say that probably the agreement between raters was only fair (on a qualitative scale). Some of the reasons for fair agreement could be difficulties in understanding the task, complex biomedical sentences beyond the scope of the average worker or random answers provided by malicious workers. The cost of using an extra rater was two times higher than the workers’ revisions, and we can safely assume that the cost of domain expertise would be even higher (if not in-house). Thus, even though the inter-rater agreement is higher between the extra rater plus expert, we can only reflect on the cost-benefit concerning MTurk workers upon further evaluation of performance, such as training the RE deep learning systems with the resulting datasets.

It was particularly interesting to have an extra rater (on-site) to express doubts while performing the task. Some of these doubts could be the ones that the workers had, while others we considered to be beyond their expertise. One of the most prominent problems for our on-site rater was if the gene entities tagged were, in fact, gene entities or their protein products that frequently share the same names. One could argue that a relation between a gene product and a human phenotype implies a relation between a gene and a human phenotype. Nonetheless, the extra rater considered that these relations hold even if the mention was of a protein and not the gene if this distinction was not clear by the sentence (only when reading the abstract or full-text article) or if the gene name was not capitalized. This particular problem was not one that a person not familiarized with biochemistry-related domains would have. However, assessing if an abbreviation that is used both as a gene name and in other biomedical topics (e.g. disease abbreviation) is a gene is a transversal problem to both the workers and our extra rater on-site.

The difficulties that our extra rater experienced are evident by the inter-rater agreement between this rater and our domain expert. On a qualitative scale, that ranges from poor agreement (<0) to almost perfect agreement (>0.81), it reflects a substantial agreement (0.61–0.80). One example sentence where they disagreed was:

While examining pedigrees of JEB patients with **LAMA3** mutations, we observed that heterozygous carriers of functional null mutations displayed subtle enamel pitting in the absence of **skin fragility** or other JEB symptoms (PMID:27827380)

where the domain expert considered a true relation and the extra rater a false relation; this happens because the relation is one of negation (absence), which often confuses non-experts with being false. However, an implication of relation of any sort is a true relation that can be classified as positive or negative. This confusion is also noticeable by the diversity in the workers’ answers for this sentence (four classified as true, two as false and one as an error).

### Corpus statistics

Table 4 presents the final numbers, both in total count and percentage for each task. For Task 2, we considered the majority consensus described previously and the domain expert numbers separately. All percentage points are referring to the original totals for Tasks 1 and 2. For instance, the expert excluded percentage is 32.02% (765 relations) of the original total for Task 2 (2389 relations). The totals always refer to the sum of the number of true and false relations.

**Table 4.** The original and final numbers both in total count and percentage, for Tasks 1 and 2, of true, false, excluded and total relations, considering the majority consensus and the domain expert numbers separately

Dataset		Relations			
		True	False	Excluded	Total
Task 1 (70%)	Original	1751 (31.41%)	3823 (68.59%)	–	5574 (100%)
	Amazon workers	4220 (75.71%)	283 (5.08%)	1071 (19.21%)	4503 (80.79%)
Task 2 (30%)	Original	729 (30.51%)	1660 (69.49%)	–	2389 (100%)
	Amazon workers + extra rater (on-site) (after reaching consensus)	1179 (49.35%)	613 (25.66%)	597 (24.99%)	1792 (75.01%)
	Expert	1281 (53.62%)	343 (14.36%)	765 (32.02%)	1624 (67.98%)

From analyzing Table 4, what becomes immediately evident is the inversion between the number of true and false relations from the original datasets to the Amazon crowdsourced datasets. These final numbers demonstrate quite clearly that most relations described in the original PGR dataset as false were, in fact, true. This inversion can be due to how the PGR dataset was built, using a gold standard knowledge base of human PGRs. At the time of the dataset creation, this knowledge base was quite incomplete, since, for instance, if a child ontological term had a relation with a gene, its immediate parent would not necessarily share the same relation, which should be explicit. Thus, these parent concepts in PGR relations would always hold false. The inversion can help populate the knowledge base with more general concepts and reinforce that true relations are generally more trustworthy than false ones within the original PGR dataset, as it is safer to prove a positive than a negative.

The column excluded represents pre-annotated NER or sentence format errors independently identified by Amazon workers (for both tasks) plus the extra rater and the expert (for Task 2). Table 4 shows that understanding the difference between an annotation error (excluded) and a false relation requires more expertise than the one that MTurk provides, and inexperienced raters have (even if in the field). Thus, we need expert knowledge to differentiate between false relations and an annotation error, such as in the following annotation error example:

We show that the **miR**-106b-25 cluster upregulates NOTCH1 in multiple breast **cancer** cell lines, representing both estrogen receptor (ER +) and triple negative breast cancer (TNBC) through direct repression of the E3 ubiquitin ligase, NEDD4L. (PMID:29 662 198)

where the workers had difficulties accessing that miR just by itself is not a gene entity, but stands for microRNA genes (a large group of genes).

We consider the third release of the PGR dataset as the revised dataset by Amazon workers for Task 1 plus the revised dataset by the domain expert for Task 2. Table 5 condenses the final numbers, considering abstracts, phenotype and gene annotations, true, false, and total relations. It is necessary to highlight that we did not consider NER annotations not participating in relations.

**Table 5.** The number of abstracts, phenotype and gene annotations, and true, false and total relations for the third release of the PGR dataset consisted of the revision of the Amazon workers (Task 1) plus domain expert revision (Task 2)

	Annotations		Relations		
Abstracts	Phenotype	Gene	True	False	Total
1921	1943	2207	5501	626	6127

**Table 6.** Precision, recall, *F*-measure and accuracy of the application of the PGR dataset (original, new and combinations between the two) to the BiOnt and BioBERT systems

Method		Precision	Recall	*F*-measure	Accuracy
BiOnt	PGR original	0.8140	0.3070	0.4459	0.4821
	Amazon Task 1 (train) + PGR original (test)	0.7000	0.9825	0.8175	0.7024
	Amazon Task 1 (train) + Amazon/extra-rater consensus Task 2 (test)	0.6810	0.9670	0.7992	0.6726
	Amazon Task 1 (train) + Expert Task 2 (test)	**0.8142**	0.9721	**0.8861**	**0.7989**
	Amazon/extra-rater consensus Task 2 (train) + PGR original (test)	0.6880	0.8509	0.7608	0.6369
	Expert Task 2 (train) + PGR original (test)	0.6894	**0.9737**	0.8072	0.6845
BioBERT	PGR original	**0.8542**	0.3445	0.4910	0.5143
	Amazon Task 1 (train) + PGR original (test)	0.6744	0.9856	0.8000	0.6775
	Amazon Task 1 (train) + Amazon/extra-rater consensus Task 2 (test)	0.6700	0.9763	0.7946	0.6680
	Amazon Task 1 (train) + Expert (test)	0.8103	**0.9906**	**0.8915**	**0.8096**
	Amazon/extra-rater consensus Task 2 (train) + PGR original (test)	0.7315	0.9160	0.8134	0.7143
	Expert Task 2 (train) + PGR original (test)	0.7857	0.8319	0.8082	0.7314

The highest scores for each metric are presented in bold.

### Deep learning impact

Table 6 presents the performance of both the original PGR dataset and the crowdsourced Amazon dataset, and combinations between the two, on the BiOnt (25) and BioBERT (version 1.1) (26) systems, in terms of precision, recall, *F*-measure and accuracy. Each experiment identifies the method and dataset employed (both for training and testing), referring to either Task 1 or Task 2 data. To assess the dataset performance (before and after crowdsourcing) when applied to deep learning systems, we used the authors’ suggested parameters. The only exception to the default parameters, since we had a class imbalance, was to add a class weight of 5 to the label false to both systems (the full multiplier to balance was ∼14.9 for the Task 1 dataset). The full multiplier results from dividing the percentage of true relations by the percentage of false relations for the training dataset. For the class weight, we chose a number between 1 and the full multiplier, which is usually the standard practice (42), to maintain a more accurate representation of the natural unbalance between labels when applying the models to real-world data. Using this class weight translates to treating every training instance with the label false as five instances of the label true, meaning that we assign a higher value to these instances in the loss function. Hence, the loss becomes a weighted average, where the weight of each sample is specified by the class weight and its corresponding class, providing a weight or bias for each output class. To achieve this, we had to alter the loss function of the BioBERT system to allow class weights.

The deep learning systems’ performance is quite similar, with BioBERT achieving slightly better results. In both systems, the performance of the new PGR dataset (through MTurk crowdsourcing) was superior to the one of the original PGR dataset, with a slight decrease in precision but a considerable gain in recall. We chose to include the accuracy metric to consider the ability to recognize true negatives (due to the class imbalance). Overall, the best performance was the Amazon MTurk (Task 1) as training corpus and the expert (Task 2) as test corpus. This performance can be due to the amount of available training data in Task 1 and the more reliable test set from the domain expert. The PGR original test set underperformed probably due to its small size, which was not representative of the data (260 relations). Also, other experiences with using the majority consensus (Task 2) and the expert (Task 2) as training sets showed that these smaller corpora also hold the ability to train a model. We achieved an increase in the average *F*-measure of 0.3494, taking into account all the experiences concerning the original PGR dataset. That is, considering the difference between the average *F*-measure (0.8179) for both deep learning methods (excluding the original PGR dataset) and the average *F*-measure (0.4685) for the original PGR dataset performance on both deep learning methods. We used the default parameters for both systems as a first pass for feasibility, achieving the range of results expected, as stated in the original articles supporting those systems. However, as future work, these parameters can be tuned not only for these tasks but to similar ones using these systems.

For the same test set (‘PGR original’), BiOnt performs better with a higher number of instances (‘Amazon Task 1’) than with fewer instances with a higher number of workers per HIT (‘Amazon/extra-rater consensus Task 2’). Whereas for BioBERT, which can perform better with less training instances, higher quality training instances perform better than a sizable number of instances. However, the differences are minimal, which implicates that when choosing quality versus quantity, the focus can be on what is more cost-effective for the task at hand, considering equivalence between 30% of the dataset reviewed by seven workers and 70% reviewed by one worker.

Table 6 also showed that a low inter-rater agreement (‘Amazon/extra-rater consensus Task 2’) implicates a significant decrease in performance for the same model compared with the domain expert test data (‘Expert Task 2’). Regardless, both systems can learn and effectively rate the ‘Amazon/extra-rater consensus Task 2’ unseen data, even at a lower precision. It is possible that the metrics used to estimate the inter-rater agreement, stated in Table 3, do not entirely reflect the MTurk set up involving the same workers doing multiple HITs. Thus, this detail justifies the difference in performance not being higher as expected due to the inter-rater agreement differences.

Our extra rater’s work had a two times superior cost than the revisions done by MTurk workers. Since our domain expert was in-house, we cannot make a proper comparison between his cost and the MTurk platform, but we can assume that would be at least the same as the extra rater, if not superior. Therefore, it is possible to state that the benefit of using MTurk, even with all its caveats, is superior cost-wise. It also takes into account the low availability of experts for some domains. With enough data, it is possible to achieve satisfactory results at a fraction of a price. However, it all depends on the budget and time available, as domain expertise knowledge remains superior.

## Conclusion and future directions

This work describes our proposal for a complete pipeline for RE crowdsourcing. The pipeline generated an openly available new release of the PGR dataset and domain expert revision into 30% of the original dataset. Additionally, we assessed MTurk workers’ performance by comparing them to an extra rater on-site and a domain expert. Moreover, we applied the new dataset as training data in two state-of-the-art deep learning systems (BiOnt (25) and BioBERT (26)) to measure the usefulness of the annotations. This study showed that it is possible to use the crowd’s wisdom to improve existing silver standard datasets since, in our case, it was able to exclude previous annotation errors (16.46%) and modify wrongly labeled relations. This improvement had a significant impact on model training since we had a 0.3494 average increase in *F*-measure, taking into account all the experiences when comparing it with the original PGR dataset. This work also showed that a lower inter-rater agreement does implicate a decrease in performance for the same model. However, the cost-benefit of using MTurk versus expert domain revision can still justify the use of the platform, as well as access to domain experts.

Regarding future work, it will be interesting to improve on the existing pipeline by providing different guidelines and assess if that would make a difference in performance. We would like to be able to validate workers through MTurk, for example, to discard workers of malicious intent or that do not meet with a specified threshold. Also, we could differentiate between what constitutes a false and a negative relation (28). To solve the lack of domain expertise of MTurk workers, we could create a specialized crowdsourcing platform for the RE biomedical field, similar to the one developed by the company Unbabel that focuses on translation (43), as well as other biomedical crowdsourcing projects (3, 44). Finally, we could apply the same methods to datasets from other biomedical domains and assess performance differences.

## Supplementary Material

baaa104_Supp
