# Supplementary material for: A hybrid approach toward biomedical relation extraction training corpora: combining distant supervision with crowdsourcing
Source: Database (Oxford). 2020 Dec 1;2020:baaa104. doi: 10.1093/database/baaa104 (PMC7706181; doi:10.1093/database/baaa104)
Supplement: baaa104_Supp [file baaa104_supp.zip › paper_database_suplementary_material.docx]

**Supplementary Material**

Supplementary Material Figure 1 Final Position

Supplementary Material Figure 1. The guidelines, in the form of examples of answers to different annotations, presented to the workers.
