# Supplementary figures and images for: A hybrid approach toward biomedical relation extraction training corpora: combining distant supervision with crowdsourcing
Source: Database (Oxford). 2020 Dec 1;2020:baaa104. doi: 10.1093/database/baaa104 (PMC7706181; doi:10.1093/database/baaa104)

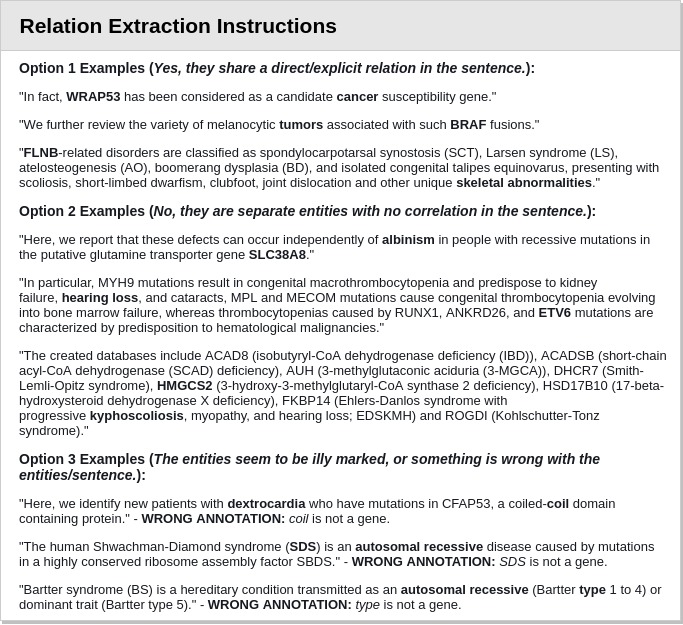

Supplement: baaa104_Supp [file baaa104_supp.zip › supplementary_material_figure_1.jpg]
